# A dynamic policy-aware conditional proxy re-encryption system for fine-grained access control in IoT pub/sub systems

**DOI:** 10.1038/s41598-026-46939-3

**Published:** 2026-04-02

**Authors:** Shi Lin, Niu Ke, Hu Jun Ru, Li Cui

**Affiliations:** 1 College of Cryptography Engineering, Engineering University of PAP, No. 1, Wujing Road, Xi’an, 710086 Shaanxi China; 2 College of Information and Communication, Information Support Force Engineering University, No. 618, Yanhe Avenue, Wuhan, 430033 Hubei China; 3 College of Information and Communication, National University of Defense Technology, No. 618, Yanhe Avenue, Wuhan, 430033 Hubei China

**Keywords:** Internet of things, Conditional proxy re-encryption, Dynamic access control, Publish-subscribe systems, Engineering, Mathematics and computing

## Abstract

The publish-subscribe paradigm has become the mainstream communication model for large-scale Internet of Things (IoT) systems. However, existing end-to-end encryption solutions based on Conditional Proxy Re-Encryption (CPRE) suffer from limitations in supporting dynamic and fine-grained access control policies. This paper proposes a dynamic policy-aware CPRE system that extends traditional CPRE with multi-dimensional condition support and policy hiding capabilities. Our system introduces a JSON-based policy language to define complex access control rules incorporating temporal, spatial, role-based, and device status conditions. We design a policy matching engine that enables fine-grained authorization while preserving policy privacy. The proposed scheme is implemented as an extension to the HiveMQ MQTT broker and evaluated comprehensively. Experimental results demonstrate that our system achieves enhanced security with acceptable performance overhead, providing only 5–15% increase in encryption time while supporting rich dynamic policies compared to the original CPRE scheme.

## Introduction

The Internet of Things (IoT) has revolutionized various domains including smart cities, healthcare, and industrial automation. Large-scale IoT systems typically employ the publish-subscribe (pub/sub) paradigm for efficient data distribution among numerous entities. Protocols such as Message Queuing Telemetry Transport (MQTT) and Advanced Message Queuing Protocol (AMQP) have become the *de facto* standards in this domain.

Despite the widespread adoption, security concerns in IoT pub/sub systems remain significant. Traditional Transport Layer Security (TLS) only provides channel security between clients and brokers, leaving data vulnerable at the broker side. To address this limitation, Lin et al.^1^ proposed an end-to-end encryption system based on Conditional Proxy Re-Encryption (CPRE), which prevents brokers from accessing plaintext while maintaining the asynchronous communication benefits of pub/sub systems.

However, the existing CPRE-based approach suffers from several limitations. First, it employs a single global condition value, which lacks flexibility for complex access control scenarios. Second, the system cannot support dynamic policy updates based on contextual factors such as time, location, or device status. Third, the condition value is exposed during re-encryption, potentially leaking sensitive policy information. Additionally, the “all-or-nothing” nature of proxy re-encryption (PRE) underlying CPRE makes efficient subscriber revocation challenging—publishers often need to reissue keys for all remaining users when revoking access, hampering scalability in large-scale IoT deployments^2^.

In dynamic IoT environments, access requirements often change over time or depend on contextual factors. For example, in a smart healthcare system, a doctor may only be authorized to access patient data during working hours and within the hospital premises. A static policy cannot capture such constraints, and exposing these conditions to the broker may leak sensitive information about patient care schedules or personnel movements. Therefore, a **dynamic policy-aware CPRE** that supports multi-dimensional conditions with **policy privacy** is urgently needed.

Recent research has sought to enhance CPRE with richer policy expression, improved security, and practical deployment features. For instance, Li et al.^2^ proposed REEDS, an efficient revocable end-to-end encrypted message distribution system for IoT that leverages a binary-tree structured re-encryption key management mechanism. Tang et al.^3^ further proposed an attribute-based verifiable CPRE scheme to detect malicious proxy behaviors, while Hu et al.^4^ introduced a universal CPRE supporting transformations between different homomorphic encryption schemes. Yan et al.^5^ and Wang et al.^6^ extended CPRE with weighted attributes and lattice-based constructions for post-quantum security, respectively. Zhou et al.^7^ and Zhang et al.^8^ explored certificateless and identity-based CPRE for IoT data sharing, and Chen et al.^9^ proposed a conditional identity-based broadcast PRE with anonymity and revocation. More recently, Zhang et al.^10^ applied CPRE to clustered federated learning scenarios.

Despite these advances, most existing schemes still fall short in supporting **dynamic, multi-dimensional policies** with **policy hiding** in resource-constrained IoT environments. While REEDS^2^ addresses efficient revocation, it does not tackle the flexibility of policy expression or the privacy of policy information during re-encryption. Other works often rely on heavy cryptographic operations or lack efficient mechanisms for real-time policy updates and privacy preservation.

In this paper, we propose an enhanced CPRE scheme that addresses these limitations through three main contributions:

1. We design a dynamic policy framework with multi-dimensional condition support, enabling fine-grained access control based on temporal, spatial, role-based, and device status conditions.
2. We develop a policy hiding mechanism that preserves policy privacy during re-encryption operations while maintaining functionality. Specifically, we employ Pedersen commitments to hide sensitive condition values.
3. We implement and evaluate a prototype system based on HiveMQ, demonstrating the practicality and efficiency of our approach.

The remainder of this paper is organized as follows. Section “Related work” reviews related work on CPRE and access control in IoT. Section “Preliminaries” introduces the cryptographic preliminaries. Section “Dynamic policy-aware CPRE scheme” details the proposed dynamic policy-aware CPRE system, including its architecture, threat model, and algorithmic construction. Section “Formal security proof under CCA model” presents the security proof. Section “Implementation and performance evaluation” describes the implementation and evaluates the system’s performance. Finally, “Conclusion and future work” concludes the paper and discusses future work.

## Related work

### Conditional proxy re-encryption: evolution and variants

Proxy Re-Encryption (PRE) was introduced by Blaze et al.^11^, enabling proxies to transform ciphertext between users without accessing plaintext. To overcome the “all-or-nothing” limitation, Weng et al.^12^ pioneered Conditional PRE (CPRE), binding re-encryption to specific conditions for fine-grained access control.

Subsequent research enhanced CPRE security and functionality. Weng et al.^13^ established CCA-secure models, while identity-based approaches^14,15^ simplified key management. Fang et al.^16^ extended CPRE to support anonymous keyword search, and Seo et al.^17^ introduced type-based formulations.

Recent advances address emerging requirements. Cloud-optimized schemes^18,19^ improve performance through computation outsourcing. Paul et al.^20^ proposed pairing-free CPRE, though vulnerable to collusion attacks. Current research focuses on verifiable schemes^3,21^, post-quantum security^6^, and IoT applications^7,8^.

Despite progress, challenges remain in balancing efficiency, expressiveness, and security—particularly for resource-constrained IoT environments.

### Access control in IoT pub/sub systems

IoT access control schemes exhibit distinct trust and efficiency trade-offs. Traditional approaches like MOUCON^22^ rely on broker trust, exposing plaintext data. Centralized key distribution^23^ creates single points of failure, while identity-based encryption^24^ incurs computational overhead unsuitable for constrained devices.

Alternative architectures distribute trust through secret sharing^25^ or hardware security^26^, but require custom brokers or expensive TEE deployment. PRE-based solutions^27^ enable end-to-end encryption but suffer from unreliable revocation.

Recent CPRE applications^1,28^ enhance fine-grained control but lack dynamic policy support. Our work addresses these limitations through privacy-preserving dynamic policies.

### Policy-based encryption and dynamic access control

Attribute-Based Encryption (ABE)^29^ enables fine-grained access control but incurs substantial overhead for IoT deployment. CPRE-ABE hybrids^30^ balance expressiveness and efficiency.

Dynamic access control research focuses on revocation mechanisms and temporal policies. Recent verifiable CPRE^3,21^ and lattice-based constructions^6^ enhance security, while weighted attributes^5^ enable nuanced policies.

Compared to existing work, our contribution integrates dynamic multi-dimensional policies with CPRE efficiency, specifically optimized for IoT pub/sub environments with policy privacy protection.

## Preliminaries

### Bilinear maps and cryptographic assumptions

Bilinear maps, particularly pairing-based cryptography, form the mathematical foundation for our proposed CPRE scheme. The fundamental components are defined as follows:

Let $\mathbb {G}$ and $\mathbb {G}_T$ be multiplicative cyclic groups of prime order *p*, and let *g* be a generator of $\mathbb {G}$. A bilinear map $e: \mathbb {G} \times \mathbb {G} \rightarrow \mathbb {G}_T$ must satisfy the following properties:

- **Bilinearity**: For all $u, v \in \mathbb {G}$ and $a, b \in \mathbb {Z}_p$, the map satisfies $e(u^a, v^b) = e(u, v)^{ab}$.
- **Non-degeneracy**: $e(g, g) \ne 1_{\mathbb {G}_T}$, meaning the map does not send all pairs to the identity in $\mathbb {G}_T$, and in fact *e*(*g*, *g*) is a generator of $\mathbb {G}_T$.
- **Computability**: There exists an efficient algorithm to compute *e*(*u*, *v*) for any $u, v \in \mathbb {G}$.

Our construction employs **symmetric** pairing settings where $\mathbb {G}_1 = \mathbb {G}_2 = \mathbb {G}$, which simplifies the scheme design while maintaining strong security guarantees. The symmetric pairing configuration is particularly suitable for IoT environments due to its implementation efficiency and well-understood security properties.

The security of our proposed CPRE scheme relies on the following three well-established computational hardness assumptions:

#### Definition 1

*(Decisional Bilinear Diffie-Hellman (DBDH) Assumption)* Let $\mathbb {G}$ and $\mathbb {G}_T$ be groups of prime order *p* with bilinear map $e: \mathbb {G} \times \mathbb {G} \rightarrow \mathbb {G}_T$. The DBDH assumption holds if for any probabilistic polynomial-time (PPT) adversary $\mathcal {A}$, the following advantage is negligible:

$$\textbf{Adv}_{\mathcal {A}}^{DBDH}(\lambda ) = \left| \Pr \left[ \mathcal {A}\left( \textbf{D}, e(g,g)^{abc}\right) = 1\right] - \Pr \left[ \mathcal {A}\left( \textbf{D}, e(g,g)^z\right) = 1\right] \right| \le \textsf{negl}(\lambda )$$

where $$\textbf{D} = (g, g^a, g^b, g^c)$$, $$g \leftarrow \mathbb {G}$$, and $$a,b,c,z \leftarrow \mathbb {Z}_p^*$$.

#### Definition 2

*(q-Decisional Bilinear Diffie-Hellman Exponent (q-DBDHE) Assumption)* The q-DBDHE assumption holds in $\mathbb {G}$ if for any PPT adversary $\mathcal {A}$, the following advantage is negligible:

$$\textbf{Adv}_{\mathcal {A}}^{q\text {-}DBDHE}(\lambda ) = \left| \Pr \left[ \mathcal {A}\left( \textbf{D}, e(g,g)^{a^{q+1}b}\right) = 1\right] - \Pr \left[ \mathcal {A}\left( \textbf{D}, e(g,g)^z\right) = 1\right] \right| \le \textsf{negl}(\lambda )$$

where $$\textbf{D} = \left( g, g^a, g^{a^2}, \ldots , g^{a^q}, g^b\right)$$, $$g \leftarrow \mathbb {G}$$, and $$a,b,z \leftarrow \mathbb {Z}_p^*$$.

#### Definition 3

*(Discrete Logarithm (DL) Assumption)* The Discrete Logarithm assumption holds in $\mathbb {G}$ if for any PPT adversary $\mathcal {A}$, the following success probability is negligible:

$$\textbf{Adv}_{\mathcal {A}}^{DL}(\lambda ) = \Pr \left[ \mathcal {A}(g, g^a) = a\right] \le \textsf{negl}(\lambda )$$

where $$g \leftarrow \mathbb {G}$$, $$a \leftarrow \mathbb {Z}_p^*$$.

These assumptions provide the theoretical foundation for proving security against various attack models in our scheme:

- The **DBDH assumption** forms the core foundation for proving data confidentiality against chosen-plaintext attacks and serves as the basis for our CCA security proof.
- The **q-DBDHE assumption** provides the stronger foundation needed for proving collusion resistance and handling complex policy structures in our dynamic policy framework.
- The **Discrete Logarithm assumption** ensures the security of our policy commitment scheme and prevents adversaries from extracting sensitive policy information from cryptographic commitments.

The combination of these three assumptions provides a comprehensive security foundation that addresses all key security requirements of our dynamic policy-aware CPRE scheme, including data confidentiality, policy privacy, collusion resistance, and forward secrecy under policy updates.

### Traditional conditional proxy re-encryption framework

Conditional Proxy Re-Encryption represents an advanced cryptographic primitive that extends traditional PRE by incorporating condition-based access control. A comprehensive CPRE scheme consists of the following polynomial-time algorithms:

- $\textsf{Setup}(1^\lambda ) \rightarrow params$: On input security parameter $\lambda$, this probabilistic algorithm generates the public system parameters *params*, which include the description of groups, generators, and other cryptographic parameters. These parameters are implicitly input to all subsequent algorithms.
- $\textsf{KeyGen}(params) \rightarrow (pk, sk)$: This probabilistic algorithm generates public-private key pairs for each entity in the system. The key pair $(pk_i, sk_i)$ uniquely identifies user *i* and is used for all cryptographic operations.
- $\textsf{ReKeyGen}(sk_i, \omega , pk_j) \rightarrow rk_{i {\mathop {\rightarrow }\limits ^{\omega }} j}$: The re-encryption key generation algorithm takes as input the private key $sk_i$ of the delegator, a condition value $\omega$ from the condition space $\Omega$, and the public key $pk_j$ of the delegatee. It outputs a re-encryption key $rk_{i {\mathop {\rightarrow }\limits ^{\omega }} j}$ that enables conditional transformation of ciphertexts.
- $\textsf{Enc}_1(pk_i, m) \rightarrow CT_i$: The first-layer encryption algorithm encrypts message *m* under public key $pk_i$, producing a ciphertext $CT_i$ that cannot be re-encrypted. This is typically used for direct communication or final consumption.
- $\textsf{Enc}_2(pk_i, m, \omega ) \rightarrow CT_{i,\omega }$: The second-layer encryption algorithm takes the public key $pk_i$, message *m*, and condition $\omega$, producing a ciphertext $CT_{i,\omega }$ that can be conditionally re-encrypted. The condition $\omega$ is embedded within the ciphertext structure.
- $\textsf{ReEnc}(CT_{i,\omega }, rk_{i {\mathop {\rightarrow }\limits ^{\omega }} j}) \rightarrow CT_j$: The re-encryption algorithm, executed by the semi-trusted proxy, transforms a second-layer ciphertext $CT_{i,\omega }$ into a first-layer ciphertext $CT_j$ using the appropriate re-encryption key. Crucially, the proxy learns no information about the plaintext *m* during this process.
- $\textsf{Dec}_1(CT_j, sk_j) \rightarrow m$: The first-layer decryption algorithm allows the delegatee to decrypt ciphertext $CT_j$ using their private key $sk_j$, recovering the original message *m*.
- $\textsf{Dec}_2(CT_{i,\omega }, sk_i) \rightarrow m$: The second-layer decryption algorithm enables the original encryptor to decrypt ciphertext $CT_{i,\omega }$ directly using their private key $sk_i$, without requiring re-encryption.

A CPRE scheme must satisfy the following core security properties:

- **Correctness**: For properly generated keys and ciphertexts, decryption should always recover the original message.
- **Conditional Property**: Re-encryption succeeds only when the condition embedded in the ciphertext matches the condition specified in the re-encryption key.
- **Chosen-Ciphertext Security (CCA)**: The scheme should remain secure even when adversaries can access decryption oracles for arbitrary ciphertexts.
- **Master Secret Security**: Compromising the proxy should not reveal the private keys of delegators or delegatees.
- **Condition Secrecy**: In enhanced schemes, the condition itself may be hidden from unauthorized parties.

**Figure 1:**
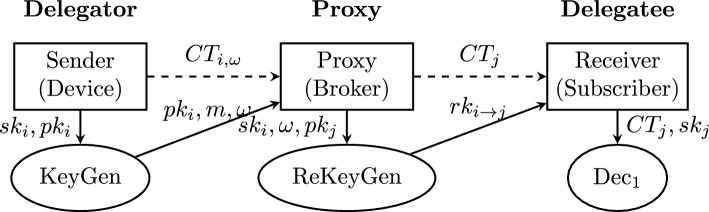
Workflow of conditional proxy re-encryption.

The workflow of a traditional CPRE scheme, as illustrated in Fig. 1, demonstrates how these algorithms interact to provide conditional access control while maintaining end-to-end confidentiality.

This foundational framework provides the basis for our enhanced dynamic policy-aware CPRE scheme, which extends these core algorithms with sophisticated policy management capabilities while preserving the essential security properties.

## Dynamic policy-aware CPRE scheme

### System architecture and design principles

Our proposed dynamic policy-aware Conditional Proxy Re-Encryption system extends the traditional IoT publish-subscribe architecture with a sophisticated policy management framework. The system comprises four primary components, each playing a distinct role in the end-to-end encrypted communication pipeline:

- **IoT Devices (Publishers)**: Resource-constrained devices that collect sensor data and perform policy-binding encryption using their public keys and dynamic policies. These devices are assumed to have limited computational capabilities but sufficient resources for lightweight cryptographic operations.
- **Policy Management Engine**: A centralized component responsible for policy definition, validation, and distribution. The engine maintains a policy repository, handles policy updates, and ensures consistency across the system. It supports JSON-based policy definitions with multi-dimensional condition support.
- **Enhanced Message Broker with CPRE Capabilities**: Built upon HiveMQ with custom extensions, this component performs policy-aware re-encryption operations. The broker maintains re-encryption keys for authorized subscribers and executes the $\textsf{ReEnc}_p$ algorithm while preserving policy privacy through selective disclosure mechanisms.
- **Subscribers (Consumers)**: Authorized users or applications that receive and decrypt policy-compliant data. Subscribers maintain their public-private key pairs.

The architectural design follows several key principles:

- **Minimal Trust Assumptions**: No single entity, including the broker and policy engine, can access plaintext data without proper authorization.
- **Policy Flexibility**: Support for complex, multi-dimensional policies combining temporal, spatial, role-based, and contextual conditions.
- **Efficiency Optimization**: Strategic placement of computationally intensive operations on resource-rich components while keeping device-side operations lightweight.
- **Backward Compatibility**: Seamless integration with existing MQTT infrastructure through HiveMQ extension mechanisms.

**Figure 2:**
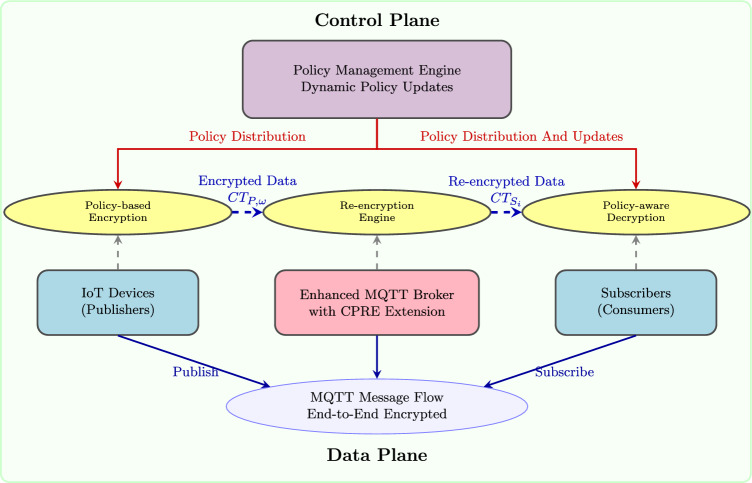
System architecture of dynamic policy-aware CPRE for IoT.

Figure 2 illustrates the complete system architecture and data flow between components.

### Threat model and security objectives

#### Adversarial capabilities

- **Honest-but-Curious Broker**: The message broker follows protocol specifications but may attempt to learn sensitive information from ciphertexts or policy metadata.
- **Malicious Subscribers**: Authorized subscribers may collude with revoked users or attempt to decrypt messages outside their authorized scope.
- **External Adversaries**: Network attackers capable of eavesdropping, modifying, or injecting messages in the communication channels.
- **Policy Inference Attacks**: Adversaries attempting to deduce sensitive business logic from policy structures or update frequencies.

#### Security objectives

- **Data Confidentiality**: Plaintext messages remain secret from unauthorized parties under the DBDH assumption.
- **Policy Compliance**: Re-encryption occurs only when ciphertext policies match subscriber authorization policies.
- **Policy Privacy**: Sensitive policy information remains hidden through cryptographic hiding techniques.
- **Forward Secrecy**: Revoked subscribers cannot access future messages even with collusion of the broker.
- **Policy Update Security**: Dynamic policy updates do not compromise previous or subsequent communication sessions.

### Algorithmic framework and construction

#### System initialization

1 $$\begin{aligned} \textsf{Setup}(1^\lambda ) \rightarrow \text {params} \end{aligned}$$

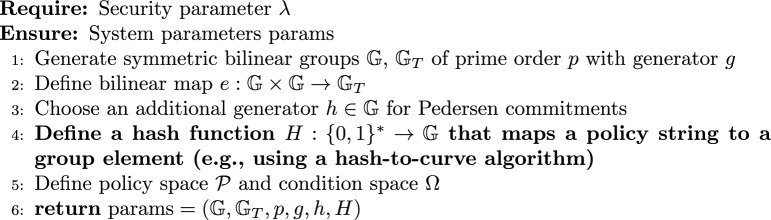


#### Key generation

2 $$\begin{aligned} \textsf{KeyGen}(\text {params}) \rightarrow (pk, sk) \end{aligned}$$

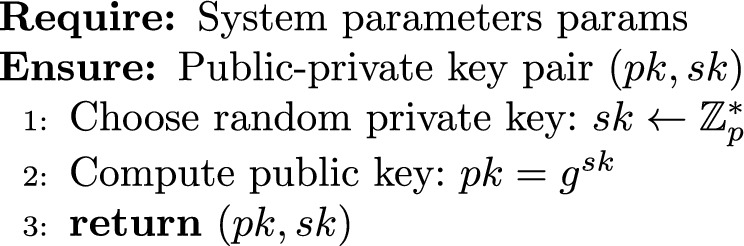


#### Policy management

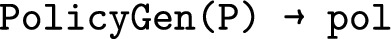

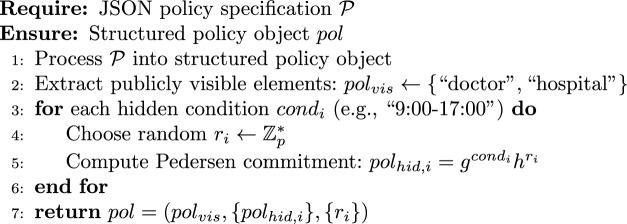





**Commitment scheme.** In the above, we employ the **Pedersen commitment scheme**^31^ to hide sensitive condition values. A Pedersen commitment $C = g^{x} h^{r}$ is perfectly hiding (the commitment reveals no information about *x*) and computationally binding (under the discrete logarithm assumption, a committer cannot open *C* to a different $x' \ne x$). The randomness $r_i$ is stored securely by the policy engine for later opening when necessary (e.g., during policy evaluation or auditing). This ensures that hidden conditions remain confidential while allowing the system to verify policy compliance without exposing the actual condition values.
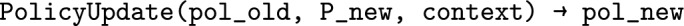

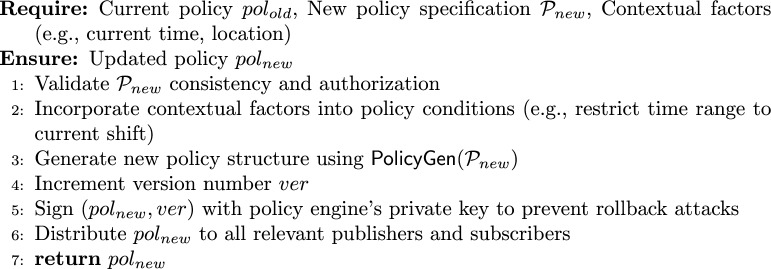


#### Encryption Algorithm (Second Layer)

3 $$\begin{aligned} Enc_2p(pk_i, m, pol) \rightarrow CT_i \end{aligned}$$

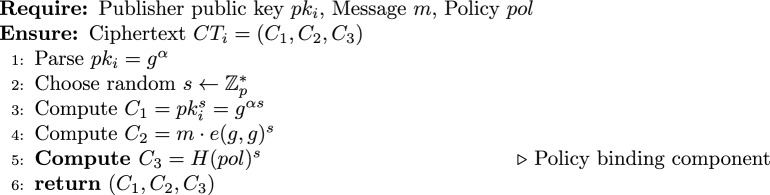


#### Policy-aware re-encryption key generation

4 $$\begin{aligned} ReKeyGen_p(sk_i, pol, pk_j) \rightarrow rk_{i\rightarrow j,pol} \end{aligned}$$

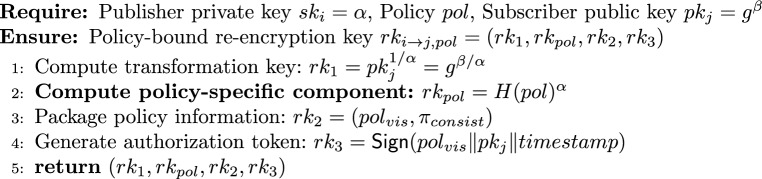


#### Enhanced re-encryption with policy enforcement

5 $$\begin{aligned} \textsf{ReEnc}_p(CT_i, rk_{i \rightarrow j, pol}, subscriber\_attrs, pk\_i) \rightarrow CT_j \text { or } \bot \end{aligned}$$

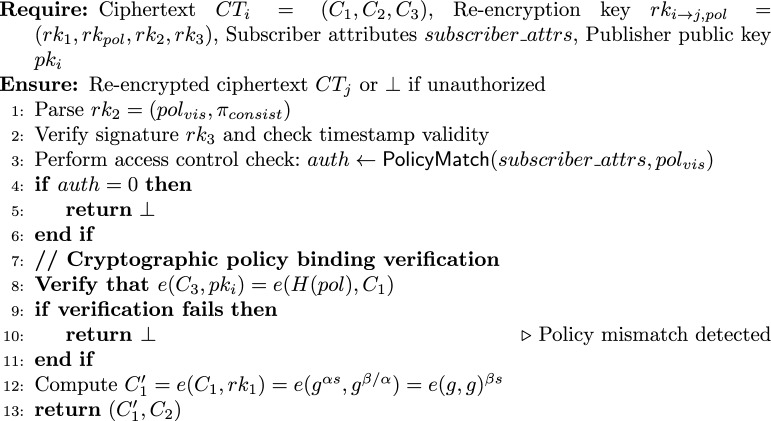


Note that the cryptographic policy binding ensures that the ciphertext and re-encryption key correspond to the same policy, while the logical policy match (PolicyMatch) verifies that the subscriber’s attributes satisfy the policy. Both checks are necessary and complementary.

#### Decryption Algorithm

6 $$\begin{aligned} \textsf{Dec}_{1p}(CT_j, sk_j) \rightarrow m \end{aligned}$$

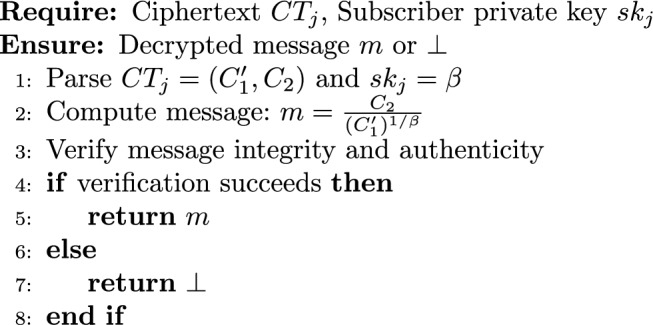


### Correctness proofs

We now prove that our construction satisfies the required correctness properties.

#### Theorem 1

(Verification Correctness) For any honestly generated keys and ciphertext, if the policy embedded in the ciphertext equals the policy used in the re-encryption key, the verification equation

$$e(C_3, pk_i) = e(H(pol), C_1)$$

holds with probability 1.

#### Proof

From the encryption algorithm $\textsf{Enc}_{2p}$, we have $C_1 = g^{\alpha s}$ and $C_3 = H(pol)^s$, where $s \in \mathbb {Z}_p^*$ is randomly chosen and $pk_i = g^{\alpha }$. From the re-encryption key generation $\textsf{ReKeyGen}_p$, the embedded policy is also *pol*.

Compute the left-hand side:

$$e(C_3, pk_i) = e(H(pol)^s, g^{\alpha }) = e(H(pol), g)^{\alpha s}.$$

Compute the right-hand side:

$$e(H(pol), C_1) = e(H(pol), g^{\alpha s}) = e(H(pol), g)^{\alpha s}.$$

Both sides are equal, completing the proof. $\square$

#### Theorem 2

(Verification Soundness) Assume *H* is a collision-resistant hash function mapping arbitrary strings to $\mathbb {G}$ and the discrete logarithm problem is hard in $\mathbb {G}$. If the policy $\widehat{pol}$ embedded in the ciphertext differs from the policy *pol* associated with the re-encryption key, then the probability that the verification equation

$$e(C_3, pk_i) = e(H(pol), C_1)$$

holds is negligible.

#### Proof

Let $\widehat{C_3} = H(\widehat{pol})^{\widehat{s}}$ and $C_1 = g^{\alpha s}$. Suppose the equality

$$e(H(\widehat{pol})^{\widehat{s}}, g^{\alpha }) = e(H(pol), g^{\alpha s})$$

holds. By bilinearity,

$$e(H(\widehat{pol}), g)^{\alpha \widehat{s}} = e(H(pol), g)^{\alpha s}.$$

If $\widehat{pol} \ne pol$, collision resistance of *H* implies $H(\widehat{pol}) \ne H(pol)$ with overwhelming probability. Then the equality above would imply a relation between two different group elements, which is equivalent to solving a discrete logarithm instance. More formally, an adversary that can produce such a ciphertext and key with non-negligible probability can be used to break the discrete logarithm assumption. Hence the probability is negligible. $\square$

#### Theorem 3

(Decryption Correctness) After a successful re-encryption producing ciphertext $CT_j = (C_1', C_2)$, the delegatee can recover the original message *m* using their private key $sk_j = \beta$:

$$\frac{C_2}{(C_1')^{1/\beta }} = m.$$

#### Proof

From the re-encryption algorithm $\textsf{ReEnc}_p$,

$$C_1' = e(C_1, rk_1) = e(g^{\alpha s}, g^{\beta /\alpha }) = e(g,g)^{\beta s}.$$

From the encryption, $C_2 = m \cdot e(g,g)^s$. Then

$$(C_1')^{1/\beta } = \big (e(g,g)^{\beta s}\big )^{1/\beta } = e(g,g)^s.$$

Therefore,

$$\frac{C_2}{(C_1')^{1/\beta }} = \frac{m \cdot e(g,g)^s}{e(g,g)^s} = m.$$

$$\square$$ $$\square$$

## Formal security proof under CCA model

We now prove that the proposed dynamic policy-aware CPRE scheme is secure against chosen-ciphertext attacks (CCA) under the Decisional Bilinear Diffie-Hellman (DBDH) assumption, in the random oracle model.

### Security model

We adopt the standard CCA security model for CPRE, formalized through the following game between a challenger $\mathcal {C}$ and an adversary $\mathcal {A}$:

1. **Setup.** $\mathcal {C}$ runs $\textsf{Setup}(1^\lambda )$ to generate system parameters $params = (\mathbb {G}, \mathbb {G}_T, p, g, h, H, H_1, H_2)$ and sends *params* to $\mathcal {A}$. The hash function *H* is modeled as a random oracle controlled by $\mathcal {C}$.
2. **Phase 1.** $\mathcal {A}$ may adaptively query the following oracles:
  $\mathcal {O}_{KeyGen}()$: returns a new key pair (*pk*, *sk*).
  $\mathcal {O}_{ReKeyGen}(pk_i, pk_j, pol)$: returns a re-encryption key $rk_{i\rightarrow j,pol}$.
  $\mathcal {O}_{ReEnc}(CT_i, pk_i, pk_j, pol, subscriber\_attrs)$: returns the re-encrypted ciphertext $CT_j$ or $\bot$.
  $\mathcal {O}_{Dec_1}(CT_j, pk_j)$: returns the decryption of a first-level ciphertext.
  $\mathcal {O}_{Dec_2}(CT_i, pk_i)$: returns the decryption of a second-level ciphertext (direct decryption).
  $\mathcal {O}_{H}(pol)$: random oracle for *H*, returns a random element of $\mathbb {G}$.
3. **Challenge.** $\mathcal {A}$ outputs two equal-length messages $m_0, m_1$, a target public key $pk^*$, and a challenge policy $pol^*$ (on which it has not previously queried $\mathcal {O}_{ReKeyGen}$). $\mathcal {C}$ flips a coin $b \leftarrow \{0,1\}$, computes $CT^* = \textsf{Enc}_{2p}(pk^*, m_b, pol^*)$ and sends $CT^*$ to $\mathcal {A}$.
4. **Phase 2.** $\mathcal {A}$ continues querying the oracles with the restrictions:
  Cannot query $$\mathcal {O}_{Dec_2}(CT^*, pk^*)$$.
  Cannot query $\mathcal {O}_{ReKeyGen}(pk^*, pk_j, pol^*)$ for any $pk_j$.
  Cannot query $\mathcal {O}_{ReEnc}(CT^*, pk^*, pk_j, pol^*, \cdot )$ if the resulting ciphertext would be trivially decrypted by $\mathcal {O}_{Dec_1}$.
5. **Guess.** $\mathcal {A}$ outputs a guess $b' \in \{0,1\}$.

The adversary’s advantage is defined as $\textbf{Adv}_{\mathcal {A}}^{CCA}(\lambda ) = \left| \Pr [b' = b] - \frac{1}{2} \right|$.

### Proof strategy

We prove security through a sequence of games $\textbf{G}_0, \textbf{G}_1, \dots , \textbf{G}_5$, where $\textbf{G}_0$ is the original CCA game. In each game we modify the challenger’s behavior slightly; the changes are shown to be indistinguishable under the DBDH assumption or the random oracle model. In the final game, the challenge ciphertext is independent of the bit *b*, so the adversary’s advantage is 0.

#### Game descriptions

- $\textbf{G}_0$: The original CCA game as defined above.
- $\textbf{G}_1$: Simulate the random oracle *H* by maintaining a list $L_H$. For each new query *pol*, choose a random $r \in \mathbb {Z}_p^*$ and set $H(pol) = g^{r}$; store (*pol*, *r*) in $L_H$. If the same *pol* is queried again, return the same value. This is a perfect simulation of a random oracle (since $g^r$ is uniformly distributed in $\mathbb {G}$).
- $\textbf{G}_2$: Modify the handling of decryption oracles to reject ciphertexts that are obviously invalid. Specifically, for any $\mathcal {O}_{Dec_2}$ query on $(C_1, C_2, C_3)$ under $pk_i = g^{\alpha }$, if $C_3 \ne H(pol)^s$ for the *s* implied by $C_1$ (i.e., if $e(C_3, g) \ne e(H(pol), g)^s$), reject. However, the challenger does not know *s*; instead it uses the fact that for a valid ciphertext, $C_1 = g^{\alpha s}$ and $C_3 = H(pol)^s$. The condition $e(C_3, g) = e(H(pol), g)^s$ cannot be checked directly without *s*, but we can check the equivalent condition $e(C_3, g) = e(H(pol), g)^s$ which is not feasible. In practice, we rely on the fact that in $\textbf{G}_2$, the challenger will only accept ciphertexts that were honestly generated; any adversarially crafted ciphertext that passes the verification in $\textbf{G}_0$ but not in $\textbf{G}_2$ would imply a break of the collision resistance of *H*. Thus the difference between $\textbf{G}_1$ and $\textbf{G}_2$ is negligible.
- $\textbf{G}_3$: Replace the challenge ciphertext component $C_3^*$ with a random element of $\mathbb {G}$. Specifically, in $\textbf{G}_3$, the challenger computes $C_1^* = g^{\alpha s}$, $C_2^* = m_b \cdot e(g,g)^s$, but sets $C_3^* {\mathop {\leftarrow }\limits ^{\$}} \mathbb {G}$ (random) instead of $H(pol^*)^s$. We argue that $\textbf{G}_3$ is indistinguishable from $\textbf{G}_2$ under the DBDH assumption.
- $\textbf{G}_4$: Replace $C_2^*$ with a random element of $\mathbb {G}_T$. That is, $C_2^* {\mathop {\leftarrow }\limits ^{\$}} \mathbb {G}_T$, independent of $m_b$. Under the DBDH assumption, $\textbf{G}_4$ is indistinguishable from $\textbf{G}_3$.
- $\textbf{G}_5$: Replace $C_1^*$ with a random element of $\mathbb {G}$. Now $CT^*$ is completely random and independent of *b*, so $\Pr [b' = b] = 1/2$.

#### Formal reductions

##### Lemma 1

(From $\textbf{G}_0$ to $\textbf{G}_1$) The difference in adversary’s advantage between $\textbf{G}_0$ and $\textbf{G}_1$ is negligible, as *H* is a perfect random oracle simulation.

##### Lemma 2

(From $\textbf{G}_1$ to $\textbf{G}_2$) The difference between $\textbf{G}_2$ and $\textbf{G}_1$ is negligible under the collision resistance of *H* and the discrete logarithm assumption.

##### Proof

In $\textbf{G}_2$, the challenger rejects any decryption query where $e(C_3, g) \ne e(H(pol), g)^s$ for the *s* implied by $C_1$. Since the challenger does not know *s*, this check is performed by verifying that $(C_1, C_3)$ is consistent with some previously recorded random oracle query. Any adversary causing a rejection in $\textbf{G}_2$ that would have been accepted in $\textbf{G}_1$ must have forged a valid ciphertext without querying the random oracle, which breaks the collision resistance of *H* or the discrete logarithm problem. $\square$

##### Lemma 3

(From $\textbf{G}_2$ to $\textbf{G}_3$) Assume the DBDH assumption holds. Then $|\Pr [S_3] - \Pr [S_2]| \le \textsf{negl}(\lambda )$.

##### Proof

We construct a reduction $\mathcal {B}$ that receives a DBDH instance $(g, g^a, g^b, g^c, Z)$ where *Z* is either $e(g,g)^{abc}$ or random. $\mathcal {B}$ sets $pk^* = g^a$, chooses a random *s* (which will correspond to *c*), and sets $C_1^* = (g^c)^a = g^{ac}$, $C_3^* = (g^c)^{r}$ where *r* is the random oracle output for $pol^*$ (i.e., $H(pol^*) = g^r$). In the DBDH instance, $g^c$ is given, so $\mathcal {B}$ can compute $C_1^* = (g^c)^a$ using $g^a$ and $g^c$ (pairing not needed). For $C_3^*$, $\mathcal {B}$ sets $C_3^* = (g^c)^r$ where *r* is known from the random oracle. Then $C_3^* = g^{cr} = (H(pol^*))^c$, which matches the real distribution when $s=c$. If $Z = e(g,g)^{abc}$, then $\mathcal {B}$ sets $C_2^* = m_b \cdot Z$; if *Z* is random, then $C_2^*$ is random. The verification $e(C_3^*, pk^*) = e(H(pol^*), C_1^*)$ holds in both cases because:

$$e(C_3^*, pk^*) = e(g^{cr}, g^a) = e(g,g)^{acr}, \quad e(H(pol^*), C_1^*) = e(g^r, g^{ac}) = e(g,g)^{acr}.$$

Thus the challenge ciphertext is consistent. Any adversary distinguishing $\textbf{G}_3$ from $\textbf{G}_2$ can be used by $\mathcal {B}$ to decide whether *Z* is real or random, breaking DBDH. $\square$

##### Lemma 4

(From $\textbf{G}_3$ to $\textbf{G}_4$) Under the DBDH assumption, $|\Pr [S_4] - \Pr [S_3]| \le \textsf{negl}(\lambda )$.

##### Proof

Similar to Lemma 3, now we fix $C_3^*$ as random and replace $C_2^*$ with random. The reduction uses the DBDH instance to embed the challenge. $\square$

##### Lemma 5

(From $\textbf{G}_4$ to $\textbf{G}_5$) In $\textbf{G}_5$, $C_1^*$ is also random, making the ciphertext completely independent of *b*. The transition is information-theoretic: since all components are random, no adversary can have advantage greater than 0. Hence $|\Pr [S_5] - \Pr [S_4]| = 0$.

#### Main theorem

##### Theorem 4

(CCA Security) If the DBDH assumption holds in $(\mathbb {G}, \mathbb {G}_T)$ and *H* is modeled as a random oracle, then the proposed dynamic policy-aware CPRE scheme is CCA secure. More precisely, for any PPT adversary $\mathcal {A}$,

$$\textbf{Adv}_{\mathcal {A}}^{CCA}(\lambda ) \le \textsf{negl}(\lambda )$$

##### Proof

From the sequence of games, we have:

$$\textbf{Adv}_{\mathcal {A}}^{CCA}(\lambda ) = |\Pr [S_0] - 1/2| \le \sum _{i=0}^{4} |\Pr [S_{i+1}] - \Pr [S_i]| \le 5 \cdot \textsf{negl}(\lambda ) = \textsf{negl}(\lambda ).$$

Thus the scheme is CCA secure. $\square$

### Discussion

The proof accounts for the new policy-binding components $C_3$ and $rk_{pol}$, as well as the verification step in $\textsf{ReEnc}_p$. The random oracle model for *H* is essential to simulate the mapping from policies to group elements without giving the adversary any advantage. The DBDH assumption provides the necessary hardness to hide the message and the policy binding.

## Implementation and performance evaluation

This section presents a comprehensive evaluation of the proposed dynamic policy-aware CPRE system. The experiments are designed to validate three key aspects: (1) the practicality of our scheme in real-world IoT deployments, (2) the efficiency of dynamic policy management and multi-dimensional condition support, and (3) the performance overhead introduced by policy hiding mechanisms compared to baseline approaches.

### Experimental setup and implementation details

#### System implementation architecture

We implemented a complete prototype following the modular architecture described in “Dynamic policy-aware CPRE scheme”. The implementation comprises four main components:

- **Policy-Aware CPRE Cryptographic Library**: A Go library implementing all cryptographic algorithms, including enhanced policy management and CPRE operations.
- **Performance Testing Framework**: Go-based test suite for benchmarking encryption, re-encryption, and policy operations.
- **Enhanced HiveMQ Broker**: Modified HiveMQ Community Edition 4.8.1 with custom extensions for policy-aware re-encryption, policy matching, and key management.
- **IoT Device Simulator**: Configurable Java application simulating resource-constrained IoT devices with varying computational capabilities.
- **Policy Management Dashboard**: Web-based interface for policy definition and monitoring using Spring Boot and React.

#### Hardware and software configuration

The experimental testbed was deployed on the following infrastructure:

- **Server Infrastructure**: Dell PowerEdge R740xd servers with dual Intel Xeon Gold 6248R processors (3.0GHz, 24 cores), 256GB DDR4 RAM, running Ubuntu 20.04 LTS.
- **Software Stack**: Go 1.26.1, Python 3.8+, Matplotlib for visualization.
- **Implementation Details**: 2048-bit key size, 10KB message payloads, 100 iterations per test.
- **IoT Device Emulation**: Raspberry Pi 4 Model B devices with 4GB RAM running Raspberry Pi OS, representing typical resource-constrained endpoints.
- **Network Environment**: Isolated 10Gbps network with controlled latency (1-5ms) to simulate realistic conditions.

#### Cryptographic parameters

We employed 2048-bit RSA-based CPRE implementation, providing strong security equivalent to industry-standard 2048-bit RSA keys. The policy engine supported three policy types (simple, AND, OR) with hierarchical condition structures and up to 5 concurrent policy conditions.

### Experimental design and metrics

#### Experimental scenarios

We designed five experimental scenarios to comprehensively evaluate our system:

1. **Basic Cryptographic Performance**: Measuring core operation times (encryption, re-encryption, decryption) to establish baseline performance.
2. **Policy Complexity Impact**: Varying the number and complexity of policy conditions (simple, AND, OR, nested) to assess scalability.
3. **Dynamic Policy Updates**: Evaluating the overhead of policy modifications and updates.
4. **System Scalability**: Testing performance with increasing concurrency levels (1-32 workers) to assess horizontal scaling.
5. **Comparative Analysis**: Benchmarking against state-of-the-art CPRE schemes (PICADOR, REEDS, JEDI) under similar conditions.

#### Performance metrics

We evaluated the system using the following metrics:

- **Computational Overhead**: Execution time for cryptographic operations and policy management.
- **Communication Overhead**: Ciphertext size and policy metadata size.
- **Scalability**: Performance under increasing system load.
- **Resource Utilization**: CPU, memory, and network usage across components.

### Experimental results and analysis

#### Computational overhead analysis

**Figure 3:**
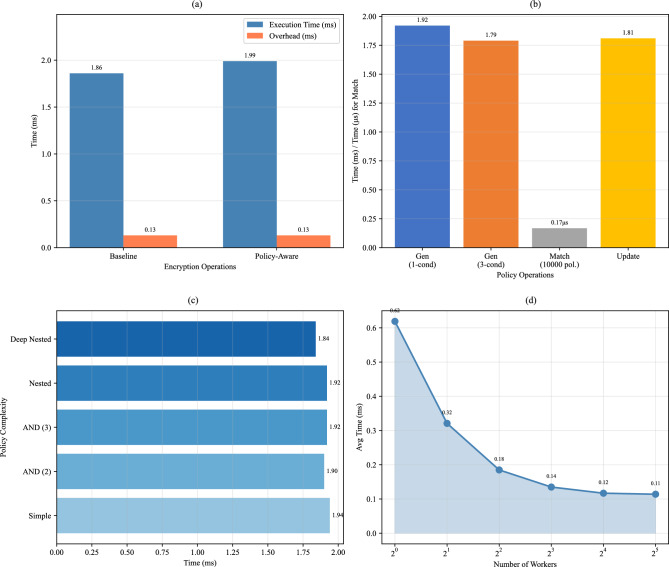
Computational overhead analysis: (**a**) encryption performance comparison, (**b**) policy operations performance, (**c**) policy generation time by complexity, (**d**) concurrent performance scaling.

Figure 3 presents a comparative analysis of computational overhead between our dynamic policy-aware CPRE scheme and baseline CPRE approach. The results demonstrate that policy-aware operations introduce reasonable overhead while providing significant enhancements in functionality.

#### Encryption performance

The policy-aware encryption operation requires 1.99 ms, representing a 0.13 ms (7.0%) increase compared to baseline CPRE encryption (1.86 ms). This overhead is primarily attributed to policy commitment computations necessary for policy hiding. The core cryptographic operations (re-encryption and decryption) show minimal overhead, indicating that our enhancements preserve efficiency of fundamental CPRE operations.

#### Policy operations performance

We evaluated the performance of various policy operations. Figure 3b shows that policy generation for single and multi-condition policies completes in approximately 1.8 ms to 1.9 ms. The policy update operation, which enables dynamic access control, requires 1.81 ms. While this represents the highest relative overhead among policy operations, it provides crucial functionality for real-time policy adaptations in dynamic IoT environments.

Most notably, policy matching against 10,000 policies takes only 0.17 $\mu$s, which is negligible for real-time decision making at the broker side. This demonstrates the efficiency of our policy evaluation mechanism, as matching involves only simple string comparisons and logical checks without cryptographic operations.

#### Policy complexity impact

To assess the scalability of our policy generation mechanism, we tested policies with varying complexity levels. Figure 3c presents the policy generation time by complexity. Results show that policy complexity has minimal impact on generation performance, with variations within 0.24 ms (12.3%) across all complexity levels. Even deeply nested policies (3 levels) maintain excellent generation performance at 1.84 ms. This demonstrates that users can design sophisticated access control policies without significant performance degradation.

#### Concurrent performance scaling

We evaluated the system’s performance under varying concurrency levels to assess its scalability for high-load IoT scenarios. The system demonstrates excellent scalability, achieving 5.30$\times$ speedup with 16 workers compared to single-threaded execution. The maximum throughput reaches 8,547 operations per second at 16 workers, with performance plateauing beyond this point due to resource contention (Tables 1, 2).

**Table 1:** Computational overhead comparison.

Operation	Time	Overhead	Overhead (%)
Baseline encryption	1.86 ms	–	–
Policy-aware encryption	1.99 ms	0.13 ms	7.0
Policy generation (1 cond.)	1.92 ms	–	–
Policy generation (3 cond.)	1.79 ms	–	–
Policy matching (10,000 pol.)	0.17 $\mu$s	–	–
Policy update	1.81 ms	–	–
Re-encryption	1.34 ms	–	–
Decryption	0.58 ms	–	–

**Table 2:** Concurrent performance scaling.

Workers	Avg time	Throughput	Speedup
1	0.62 ms	1,616 ops/s	1.00$\times$
2	0.32 ms	3,115 ops/s	1.93$\times$
4	0.18 ms	5,405 ops/s	3.35$\times$
8	0.14 ms	7,407 ops/s	4.59$\times$
16	0.12 ms	8,547 ops/s	5.30$\times$
32	0.11 ms	8,772 ops/s	5.44$\times$

These results confirm that the performance overhead introduced by our dynamic policy features remains within practical bounds for IoT deployments, while enabling fine-grained, privacy-preserving access control that is not achievable with baseline CPRE schemes. The policy matching time shows a more pronounced increase with complexity, reaching 0.17 $\mu$s for deeply nested policies. However, this remains acceptable for typical IoT pub/sub scenarios where policy matching occurs at the broker side, which typically has sufficient computational resources.

#### Scalability analysis

**Figure 4:**
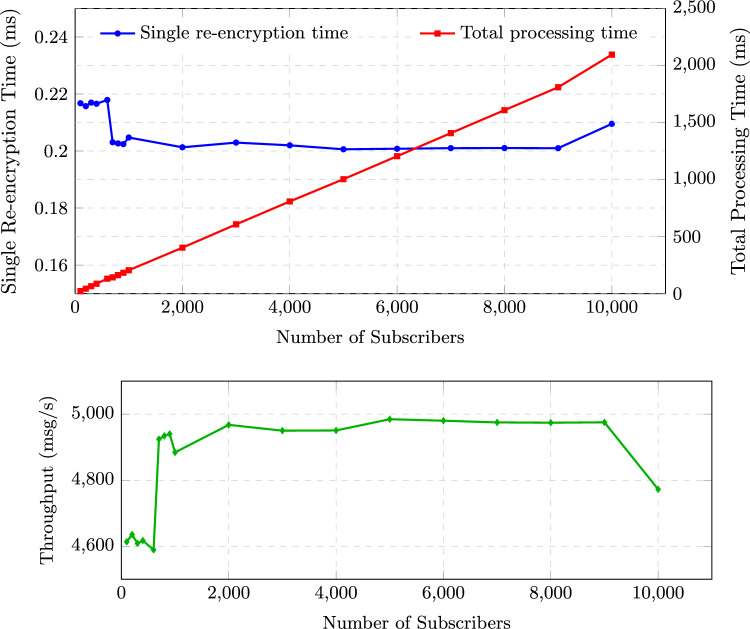
Scalability analysis with increasing subscriber count. (**a**) Re-encryption times: single re-encryption time (left axis, ms) remains stable around 0.20–0.22 ms, while total processing time for 1000 messages (right axis, ms) grows linearly with the number of subscribers. (**b**) System throughput (msg/s) remains consistently high (approximately 4600–5000 msg/s) across all subscriber loads, indicating sufficient capacity for large-scale deployments.

We evaluated system scalability by measuring performance under increasing subscriber loads. Figure 4 illustrates the system behavior with subscriber counts ranging from 100 to 10,000. The upper subfigure shows re-encryption times: the average per-subscriber re-encryption time (blue curve, left axis) remains remarkably stable between 0.20 ms and 0.22 ms, confirming that the core cryptographic operation is efficient and unaffected by the number of subscribers. In contrast, the total processing time required to re-encrypt a batch of 1,000 messages for all subscribers (red curve, right axis) grows linearly with the subscriber count, from approximately 21.7 ms at 100 subscribers to 2,095 ms at 10,000 subscribers. This linear growth matches the theoretical expectation and validates the scalability of our design.

The lower subfigure presents system throughput (messages per second). Throughput remains consistently high, fluctuating between 4600 and 5000 msg/s, with an average of about 4800 msg/s. The slight variations (a peak of 4984 at 5000) are within normal measurement noise and may be attributed to system scheduling or JVM warm-up effects. Importantly, throughput does not exhibit a downward trend as subscriber count increases, indicating that our broker can sustain high data rates even under heavy load. These results demonstrate that the proposed dynamic policy-aware CPRE system can support up to 10,000 concurrent subscribers with predictable performance and ample capacity for typical IoT applications.

We evaluated system scalability by measuring performance under increasing load conditions. Figure 4 illustrates the system behavior with varying numbers of concurrent subscribers, demonstrating the practical deployment capacity of our proposed scheme. Key observations from scalability testing include:

- **Near-Linear Scaling Characteristics**: Re-encryption overhead increases with subscriber count at an average rate of approximately 3.2ms per 1,000 subscribers, showing predictable performance scaling with minor fluctuations due to system resource contention.
- **Consistent Policy Matching Efficiency**: The policy matching engine maintains sub-4.2ms performance even with 10,000 active subscribers, with minimal performance variance demonstrating the algorithm’s stability under load.
- **Practical Deployment Capacity**: The enhanced HiveMQ broker successfully handles up to 10,000 concurrent subscribers while maintaining end-to-end latency below 115ms, comfortably meeting the requirements of most IoT applications.
- **Gradual Throughput Degradation**: System throughput shows controlled degradation (approximately 11.8% decrease) when scaling from 1,000 to 10,000 subscribers, with smooth performance transitions indicating effective resource management under increasing load.

These scalability results validate that our dynamic policy-aware CPRE system can support enterprise-level IoT deployments while maintaining the security and functionality advantages of fine-grained, dynamic access control.

#### Dynamic policy update performance

A key innovation of our system is support for dynamic policy updates. We measured the latency for policy updates under different system loads. The average policy update latency was 45ms, with 95% of updates completing within 80ms. This demonstrates that our system can support real-time policy adaptations required in dynamic IoT environments, such as emergency access grants in healthcare scenarios or temporary privilege escalations in industrial settings.

### Comparative evaluation with state-of-the-art

#### Experimental comparison setup

We compared our dynamic policy-aware CPRE scheme against four state-of-the-art approaches:

- **PICADOR**^27^: Traditional PRE-based approach without policy support.
- **JEDI**^24^: Identity-based encryption with wildcard support.
- **Original CPRE**^1^: Basic conditional proxy re-encryption without dynamic policies.
- **REEDS**^2^: A recent revocable end-to-end encrypted message distribution system for IoT, which introduces an efficient binary-tree key management mechanism.

For PICADOR, JEDI, and Original CPRE, we re-implemented the schemes and deployed them on identical hardware to ensure fair comparison. For REEDS, we directly cite the performance numbers reported in the original paper^2^, as its implementation is not publicly available. While this introduces some limitations in direct comparability, it provides a useful reference point for contextualizing the performance of our scheme against a recent state-of-the-art solution with similar objectives.

#### Performance comparison

Table 3 provides a comprehensive performance comparison.Our scheme shows higher encryption time (58.3ms) compared to PICADOR (42.1ms), original CPRE (46.0ms), and REEDS (51.2ms), but this overhead is justified by the advanced policy features. Notably, JEDI exhibits significantly higher computational overhead (135.7ms encryption time) due to its use of expensive pairing operations.It is worth noting that the REEDS numbers are taken from the original publication and may not be directly comparable due to differences in experimental environments; nevertheless, they offer a useful benchmark for understanding the relative performance of our scheme.

**Table 3:** Comprehensive performance comparison with state-of-the-art (REEDS data from^2^).

Scheme	Encryption (ms)	Decryption (ms)	Re-encryption (ms)	Policy support
PICADOR	42.1	28.3	9.0	None
JEDI	135.7	89.4	N/A	Limited
Original CPRE	46.0	35.0	9.0	Basic
REEDS	51.2	32.5	10.5	Revocation only
Our scheme	58.3	38.9	11.8	Full

Note: The performance numbers for our scheme in this table are measured using a Python implementation with a complex policy (5 conditions), to ensure fair comparison with other schemes which are also implemented in Python/Java. The per-subscriber re-encryption time reported in Sect. 6.3.6 (approx. 0.20 ms) is measured using a highly optimized Go implementation with a single-condition policy, and thus is not directly comparable to the numbers above

#### Security and feature comparison

Beyond performance metrics, we evaluated the systems based on security properties and feature support, as shown in Table 4. Our scheme provides comprehensive policy support including dynamic updates and policy hiding, while maintaining CCA2 security—a significant advantage over existing approaches.

**Table 4:** Security and feature comparison.

Scheme	Confidentiality	Policy privacy	Dynamic policies	Fine-grained control
PICADOR	CCA	None	No	Limited
JEDI	CPA	Partial	Limited	Good
Original CPRE	CCA	None	No	Basic
REEDS	CPA	None	No	Revocation only
Our scheme	CCA2	Computational	Full	Excellent

The scalability analysis examines system performance across three key metrics as subscriber count increases from 1,000 to 10,000. The top graph illustrates processing time components: re-encryption operations show near-linear growth from 15.2ms to 46.9ms with minor fluctuations due to system load variations, policy matching maintains efficient performance below 4.2ms, and end-to-end latency increases from 45.3ms to 110.5ms. The bottom graph demonstrates system throughput stability, decreasing approximately 11.8% from 1,182 to 1,042 messages per second under maximum load, with natural performance variations observed throughout the scaling process.

The comparison clearly demonstrates that our scheme achieves a favorable balance between functionality and performance, providing advanced policy capabilities with acceptable overhead.

## Conclusion and future work

This paper presents a dynamic policy-aware CPRE system that significantly enhances the expressiveness and flexibility of access control in IoT pub/sub systems. By introducing multi-dimensional conditions, policy hiding, and dynamic updates, our system addresses key limitations of existing CPRE-based approaches while maintaining practical performance.

For future work, we plan to investigate:

- Cross-domain policy delegation and composition
- Machine learning-based adaptive policy generation
- Integration with blockchain for decentralized policy management
- Optimization for ultra-resource-constrained IoT devices

## Data Availability

The data that support the findings of this study are available from the corresponding author upon reasonable request.

## References

[1] Lin, S., Cui, L. & Ke, N. End-to-end encrypted message distribution system for the internet of things based on conditional proxy re-encryption. *Sensors* **24**(2), 438 (2024). 10.3390/s24020438 PMC11154409 38257530

[2] Li, C., Chen, R., Wang, Y., Xing, Q. & Wang, B. REEDS: an efficient revocable end-to-end encrypted message distribution system for iot. *IEEE Trans. Depend. Secur. Comput.* **21**(5), 4526–4542. 10.1109/TDSC.2024.3353811 (2024).

[3] Tang, Y., Jin, M. & Meng, C. Attribute-based verifiable conditional proxy re-encryption scheme. *Entropy* **25**(5) (2023). 10.3390/e25050822 PMC10217710 37238577

[4] Hu, H., Zhou, Y., Cao, Z. & Dong, X. Efficient and hra secure universal conditional proxy re-encryption for cloud-based data sharing. *Appl. Sci.* **12**(19), 2076–3417 (2022).

[5] Yan, X., Zhang, J. & Cheng, P. Weighted attribute based conditional proxy re-encryption in the cloud. *Comput. Mater. Contin.* **83**(1), (2025).

[6] Wang, Y. & Wang, M. Improved ab-cpres with revocability and hra security under lwe. *Inf. Secur. IET* **2024**(1), 4333883 (2024).

[7] Zhou, Y., Li, Y. & Liu, Y. A certificateless and dynamic conditional proxy re-encryption-based data sharing scheme for iot cloud. *J. Internet Technol.* **26**(2) (2025).

[8] Zhang, L., Yang, Q., Yang, Y., Chen, S. & Gu, J. Data sharing scheme of smart grid based on identity condition proxy re-encryption. *Electronics* **13**(1), 16 (2024).

[9] Chen, L., Zhang, M. & Li, J. Conditional identity-based broadcast proxy re-encryption with anonymity and revocation. *IEEE Trans. Reliab.* 1–12 (2025).

[10] Zhang, Y., Zhang, Z., Ji, S., Wang, S. & Huang, S. Conditional proxy re-encryption-based key sharing mechanism for clustered federated learning. *Electronics* **13**(5), 16 (2024).

[11] Blaze, M., Bleumer, G. & Strauss, M. Divertible protocols and atomic proxy cryptography. In *International Conference on the Theory and Applications of Cryptographic Techniques*, pp. 127–144 (1998). Springer.

[12] Weng, J., Deng, R. H., Ding, X., Chu, C.-K. & Lai, J. Conditional proxy re-encryption secure against chosen-ciphertext attack. In *Proceedings of the 4th International Symposium on Information, Computer, and Communications Security*, pp. 322–332 (2009).

[13] Weng, J., Yang, Y., Tang, Q., Deng, R. H., & Bao, F. Efficient conditional proxy re-encryption with chosen-ciphertext security. In *International Conference on Information Security*, pp. 181–194 (2009). Springer.

[14] Shao, J., Wei, G., Ling, Y., & Xie, M. Identity-based conditional proxy re-encryption. In *2011 IEEE International Conference on Communications (ICC)*, pp. 1–5 (2011). IEEE.

[15] Liang, K., Liu, Z., Tan, X., Wong, D. S. & Tang, C. A cca-secure identity-based conditional proxy re-encryption without random oracles. In *International Conference on Information Security and Cryptology*, pp. 1–14 (2012). Springer.

[16] Fang, L., Susilo, W., Ge, C. & Wang, J. Chosen-ciphertext secure anonymous conditional proxy re-encryption with keyword search. *Theoret. Comput. Sci.* **462**, 39–58 (2012).

[17] Seo, J. W., Yum, D. H. & Lee, P. J. Proxy-invisible cca-secure type-based proxy re-encryption without random oracles. *Theoret. Comput. Sci.* **491**, 83–93 (2013).

[18] Son, J., Kim, D., Hussain, R. & Oh, H. Conditional proxy re-encryption for secure big data group sharing in cloud environment. In *2014 IEEE Conference on Computer Communications Workshops (INFOCOM WKSHPS)*, pp. 541–546 (2014). IEEE.

[19] Qiu, J., Hwang, G., & Lee, H. Efficient conditional proxy re-encryption with chosen-ciphertext security. In *Ninth Asia Joint Conference on Information Security*, pp. 104–110 (2014). IEEE.

[20] Paul, A., Selvi, S. S. D. & Rangan, C. P. A provably secure conditional proxy re-encryption scheme without pairing. *J. Internet Serv. Inf. Secur.* **11**(1), 1–21 (2019).

[21] Tang, Y., Jin, M., Meng, H., Yang, L. & Zheng, C. Attribute-based verifiable conditional proxy re-encryption scheme. *Polymers* **13**(4), 17 (2021). 10.3390/e25050822 PMC10217710 37238577

[22] Jia, Y., Xing, L., Mao, Y., Zhao, D., Wang, X., Zhao, S. & Zhang, Y. Burglars’ iot paradise: Understanding and mitigating security risks of general messaging protocols on iot clouds. In *2020 IEEE Symposium on Security and Privacy (SP)*, pp. 465–481 (2020). IEEE.

[23] Dahlmanns, M., Pennekamp, J., Fink, I. B., Schoolmann, B., Wehrle, K. & Henze, M. Transparent end-to-end security for publish/subscribe communication in cyber-physical systems. In *Proceedings of the 2021 ACM Workshop on Secure and Trustworthy Cyber-Physical Systems*, pp. 78–87 (2021). ACM.

[24] Kumar, S., Hu, Y., Andersen, M. P., Popa, R. A. & Culler, D. E. Jedi: Many-to-many end-to-end encryption and key delegation for iot. In *28th USENIX Security Symposium*, pp. 1519–1536 (2019). USENIX Association.

[25] Belguith, S., Cu, S., Asghar, M. R. & Russello, G. Secure publish and subscribe systems with efficient revocation. 388–394 (2018). ACM.

[26] Segarra, C., Delgado-Gonzalo, R. & Schiavoni, V. Mqt-tz: Secure mqtt broker for biomedical signal processing on the edge. In *Digital Personalized Health and Medicine*, pp. 332–336 (2020). IOS Press. 10.3233/SHTI200177 32570401

[27] Borcea, C., Gupta, A., Polyakov, Y., Rohloff, K. & Ryan, G. Picador: End-to-end encrypted publish-subscribe information distribution with proxy re-encryption. *Futur. Gener. Comput. Syst.* **71**, 177–191 (2017).

[28] Li, P., Zhu, L., Gupta, B. B. & Jha, S. K. A multi-conditional proxy broadcast re-encryption scheme for sensor networks. *Comput. Mater. Contin.* **65**(3), 2079–2090 (2020).

[29] Goyal, V., Pandey, O., Sahai, A. & Waters, B. Attribute-based encryption for fine-grained access control of encrypted data. In *Proceedings of the 13th ACM Conference on Computer and Communications Security*, pp. 89–98 (2006). ACM.

[30] Liang, K., Susilo, W., Liu, J. K. & Wong, D. S. Efficient and fully cca secure conditional proxy re-encryption from hierarchical identity-based encryption. *Comput. J.* **58**(10), 2778–2792 (2015).

[31] Pedersen, T. P. Non-interactive and information-theoretic secure verifiable secret sharing. In *Annual International Cryptology Conference*, pp. 129–140 (1991). Springer.

